# Exploring the role of sex differences in Alzheimer's disease pathogenesis in Down syndrome

**DOI:** 10.3389/fnins.2022.954999

**Published:** 2022-08-12

**Authors:** Elizabeth J. Andrews, Alessandra C. Martini, Elizabeth Head

**Affiliations:** ^1^Department of Pathology and Laboratory Medicine, University of California, Irvine, Irvine, CA, United States; ^2^Institute for Memory Impairments and Neurological Disorders, University of California, Irvine, Irvine, CA, United States

**Keywords:** amyloid beta, tau tangles, hormones, estrogen, metabolism, vascular, inflammation, aging

## Abstract

Women are disproportionately affected by Alzheimer's disease (AD), yet little is known about sex-specific effects on the development of AD in the Down syndrome (DS) population. DS is caused by a full or partial triplication of chromosome 21, which harbors the amyloid precursor protein (APP) gene, among others. The majority of people with DS in their early- to mid-40s will accumulate sufficient amyloid-beta (Aβ) in their brains along with neurofibrillary tangles (NFT) for a neuropathological diagnosis of AD, and the triplication of the APP gene is regarded as the main cause. Studies addressing sex differences with age and impact on dementia in people with DS are inconsistent. However, women with DS experience earlier age of onset of menopause, marked by a drop in estrogen, than women without DS. This review focuses on key sex differences observed with age and AD in people with DS and a discussion of possible underlying mechanisms that could be driving or protecting from AD development in DS. Understanding how biological sex influences the brain will lead to development of dedicated therapeutics and interventions to improve the quality of life for people with DS and AD.

## Background

Down syndrome (DS) is the most common cause of intellectual disability in the United States, caused by a partial or full triplication of chromosome 21 (Antonarakis et al., 2020). Within the population, virtually all people with DS will develop Alzheimer's disease (AD) neuropathology, namely amyloid-beta (Aβ) plaques and neurofibrillary tangles, by 40 years of age. This is likely due to the overexpression of the amyloid precursor protein (APP) gene on chromosome 21 (Prasher et al., 1998; Doran et al., 2017; Lott and Head, 2019). The formation of these key pathologies contribute to the onset of AD dementia, which presents around the age of 53–55 years in people with DS (Strydom et al., 2018; Lott and Head, 2019; Antonarakis et al., 2020; Fortea et al., 2021). As in DS, AD in the general population is a progressive neurodegenerative disease associated with dementia and the accumulation of amyloid-beta plaques and neurofibrillary tangles along with neuronal loss (Crews and Masliah, 2010). The most common form of AD is late onset AD (LOAD), which occurs in people over the age of 65 years (Koedam et al., 2010). The most significant risk factors for LOAD include age and the presence of an ApoE4 allele (Liu et al., 2013).

Research examining sex differences in the neurotypical population suggests a general consensus that women are at higher risk for developing LOAD (Fiest et al., 2016; Matthews et al., 2016; Podcasy and Epperson, 2016). Although heightened vulnerability to AD in women suggests a possible role for hormones in AD pathogenesis, clinical trials with hormone replacement therapy (HRT) to delay onset of dementia are inconclusive or are associated with negative effects (Podcasy and Epperson, 2016). In LOAD, women exhibit higher levels of AD pathology than men at autopsy (Oveisgharan et al., 2018), and tend to live longer with AD than their male counterparts (Tang et al., 1996; Hebert et al., 2001). In addition, positron emission tomography (PET) imaging shows that women often have significantly greater tau burden than men (Buckley et al., 2019b). One of the potential modifiers for the influence of biological sex on AD could lie in the presence of the APOE4 allele. Apolipoprotein (ApoE) is a protein involved in lipid metabolism and trafficking, which has been linked to neurodegeneration (Huang and Mahley, 2014). APOE, the gene responsible for producing ApoE, can have multiple alleles predisposing the carrier to dementia. The presence of one or two E4 alleles substantially increases risk for developing dementia, specifically AD-related dementia (Safieh et al., 2019). Female APOE4 carriers are also more likely to develop AD than are men with this allele (Altmann et al., 2014), which may contribute to differences observed in pathology between men and women.

It is also important to note that though we will refer to human females and males as “women” and “men”, this is in reference to biological sex only, and not gender. The element of gender identity also contributes to health outcomes and should be investigated to the same extent as molecular mechanisms; however, at the current time, there is a significant lack of research in gender identity and sexual orientation of people with DS. In this review, we will characterize some of the main sex differences that we propose may influence aging and AD development and progression in DS.

### Sex effects on the development of dementia in DS

Whether a similar heightened vulnerability to AD dementia in women holds true in the DS population is still under investigation. In a literature overview provided in Table 1, outcomes have been variable. Early studies included smaller cohorts of people with DS where adults with DS were seen in a clinical setting. In a study of 28 people with DS, ranging in age from 10 to 74 years, women had an earlier age of onset of dementia (Raghavan et al., 1994). This outcome was subsequently confirmed in at least 2 other studies. In a clinical cohort of 100 adults ranging in age from 35 to 79 years, women were 1.77 times more likely to develop dementia (Lai et al., 1999). Menopause occurs earlier in women with DS (Coppus et al., 2010). When focusing on post-menopausal women with DS, an increase in risk of AD was observed in a study of 85 women (over 45 years of age) and then confirmed in a larger cohort of 249 women (31–70 years) (Coppus et al., 2010; Zigman, 2013; Zhao et al., 2015). Indeed, the risk of AD in post-menopausal women can be up to 2-fold higher (Zhao et al., 2015) and may be related to lower levels of bioavailable estradiol (Schupf et al., 2006). In one of the larger cohort studies, Startin et al. also observed that women with DS had a higher rate of dementia compared to men with DS.

**Table 1:** Clinical studies addressing sex differences in dementia in Down syndrome.

**References**	**Sample** **(age in years)** **(Female/Male)**	**Title**	**Source**	**Findings**
Mhatre et al. (2021)	408 DS adults (age cog. stable: 49.2 ± 6.5; age dementia: 53.5 ± 5.2) (267 F/141 M)	The association between sex and risk of Alzheimer's disease in adults with Down syndrome	Journal of Clinical Medicine	Increased risk of AD observed in men with DS over the age of 60
Lai et al. (2020)	246 DS adults (age > 40) (95 F/151 M)	Sex differences in risk of Alzheimer's disease in adults with Down syndrome	Alzheimer's and dementia (Amst)	No link between sex and risk of AD; women with DS had longer duration of dementia
Landes et al. (2020)	9,870 DS adults (age > 18) (48.45% F)	Cause of death in adults with Down syndrome in the United States	Disabil Health J	Women with DS more likely to die due to dementia/AD and congenital heart defects
Startin et al. (2020)	602 DS adults (age 3 months−73 years) (288 F/314 M)	Health comorbidities and cognitive abilities across the lifespan in Down syndrome	J Neurodev Disord	Women with DS showed higher rates of dementia and autism; men with DS had higher rates of depression; both compared to general population
Zhao et al. (2015)	249 DS women (age 31–70)	Estrogen receptor-beta variants are associated with increased risk of Alzheimer's disease in women with down syndrome	Dementia and geriatric cognitive disorders	2-fold AD risk in post-menopausal women with DS carrying specific SNPs in ESR2
Coppus et al. (2010)	85 post-menopausal DS women (age > 45)	Early age at menopause is associated with increased risk of dementia and mortality in women with Down syndrome	Journal of Alzheimer's Disease	Women with DS experience menopause early, which is associated with increased risk of AD
Schupf et al. (2006)	119 post-menopausal DS women (age 42–59)	Bioavailable estradiol and age at onset of Alzheimer's disease in post-menopausal women with Down syndrome	Neuroscience Letters	Women with low levels of bioavailable estradiol are more likely to develop AD and to develop it earlier
Lai et al. (1999)	100 DS adults (age 35–79)	APOE genotype and gender effects on Alzheimer disease in 100 adults with Down syndrome	American Academy of Neurology	Women were 1.77 times more likely to develop dementia; no association between sex and APOE
Schupf et al. (1998)	111 DS adults (age 34–71) (50 F/61 M)	Earlier onset of Alzheimer's disease in men with Down syndrome	Neurology	Male carriers of APOE4 had earlier onset of AD
Raghavan et al. (1994)	28 DS adults (age 10–74) (11 F/17 M)	Gender differences in the phenotypic expression of Alzheimer's disease in Down's syndrome (trisomy 21).	Neuroreport	Women with DS had higher NFT burden; No significant difference in Aβ plaque burden

In contrast, other studies report opposite sex effects or no differences in risk of developing AD between men and women with DS. Schupf et al. (1998) found that there was an earlier age of onset of AD in a prospective study of men with DS who carried the ApoE4 allele. Lai et al. (2020) showed that in a clinical cohort of 246 adults with DS over the age of 40 years, there was no link between sex and risk of AD although women with DS appear to have a longer duration of dementia. Benejam et al. (2020) reported no difference in cognitive scores between men and women, suggesting similar cognitive changes with age. More recently, Mhatre et al. (2021) showed that men with DS over 60 years of age have a six times higher probability of developing dementia compared to women of the same age.

Hartley et al. (2020) reported no sex differences in rate of cognitive decline, although there were some differences observed in slightly better performance of males on tasks related to attention and episodic memory. It is important to note that Hartley, Lao, and others report on the transition from cognitively stable to AD/dementia, while other studies report differences after onset of AD. We know time is of particular importance to women with DS as they experience menopause earlier. Thus, biological sex could have a greater impact in later stages of AD and its progression. This could also be a reason as to why studies report such mixed results on male and female vulnerability in DS.

People with DS also experience several co-occurring illnesses/conditions that may also modify age of onset of dementia and may interact with the effect of sex on dementia, including hormone imbalances, vascular changes, and immune disorders (Antonarakis et al., 2020). Interestingly, in the study by Startin et al. (2020) where multiple comorbidities were also examined, not only did women with DS show higher rates of dementia but also autism, while men with DS exhibit higher rates of depression when compared to the neurotypical population. This is in contrast to findings by Dekker et al. (2021) who found no association between depression and sex in DS. There is some evidence to suggest women with intellectual disabilities may perform differently on cognitive tests when compared to their male counterparts as well. Women with general intellectual disability as well as women with DS performed better overall on coding tests, which measure for visual memory, and parallels what is seen in the general population (Kittler et al., 2004). During the duration of the study, although scores fell over time, the female advantage was preserved.

One aspect to note on sex differences is also that they may be more nuanced than previous generalizations and that sex differences may be task specific. In addition to discrepancies in cognitive outcomes, a study using mortality records from 9,870 individuals showed a significant difference in cause of death in women with DS compared to men with DS. Women with DS were more likely to die from congenital heart defects or due to AD and dementia than men (Landes et al., 2020). Additionally, a study of 1,199 individuals with DS observed that women were more likely to be considered overweight and physically inactive (Stancliffe et al., 2012). Differential observations across these studies may also be due to the type of cohort being followed (clinical vs. research), the type of study (prospective vs. incident dementia), how dementia is diagnosed and the smaller sample sizes typical of studies of people with DS.

### Sex differences with age in people with DS observed by neuroimaging studies—MRI

Magnetic resonance imaging (MRI) provides insight into brain structure, atrophy and neuronal density *in vivo*. In neurotypical and sporadic AD populations, several studies focused on sex differences using neuroimaging outcome measures. Women show lower gray and white matter volumes in a cohort of 121 cognitively normal individuals (85 women and 36 men; age 40–65), compared to men (Rahman et al., 2020). Loss in specific regions, namely the prefrontal cortex, amygdala and hippocampus, also overlap with the brain's estrogen network. Key findings also suggest a critical window of time during menopause when AD prevention may be most efficacious, given an earlier age of menopause has been associated with higher risk of dementia (Gilsanz et al., 2019). MRI studies in AD cohorts show differences in hippocampal volume in men and women, suggesting that women may be more susceptible to cognitive decline (Burke et al., 2019). However, it is important to consider that some structural differences in men and women in the neurotypical population do not reflect or suggest differences in cognitive abilities.

MRI is used to document changes in gray and white matter volume as a function of age and cognitive status in DS for several decades as it allows for a non-invasive method to asses brain volumes by region (Emerson et al., 1995; Teipel and Hampel, 2006). Previous MRI studies observe structural differences in DS brains relative to neurotypical controls, namely thicker frontal and occipitoparietal cortices and thinner motor cortex, as well as smaller hippocampus, but enlarged putamen (Annus et al., 2017). To date, there are no MRI studies in DS that directly investigate sex differences in relation to structure (Table 2). In addition to structure, MRIs can show white matter hyperintensities (WMH), which are lesions within the white matter. WMH are linked to AD as well as an increased risk of cerebrovascular events (Graff-Radford et al., 2019; Tubi et al., 2020). Interestingly, sex differences are observed in WMH, with men with DS and the APOE4 allele showing higher WMH volumes in the occipital lobe (Lao et al., 2020). This further underscores may be some APOE4 effect that differs by sex, as APOE4 has been revealed to be important to AD disease progression in DS (Bejanin et al., 2021). Given WMH indicates a higher chance of cerebrovascular events, including stroke, there could be a selective vulnerability for men with DS to develop cerebrovascular pathology with age. Though, this is based on a single study, and it is important to note other factors, such as hormonal involvement, sex may contribute to cerebrovascular differences, as we will examine in a later section. However, aside from cerebrovascular differences, neuroimaging outcome measures can provide insights into whether males or females with DS show variable ages of onset or extent of AD pathology.

**Table 2:** Literature on sex differences in neuroimaging in Down syndrome.

**References**	**Sample** **(age in years)** **(Female/Male)**	**Title**	**Source**	**Findings**	**Tau/Aβ**	**PET/MRI**
Bejanin et al. (2021)	464 DS adults (age 34–50) (214 F/250 M)	Association of apolipoprotein E ε4 allele with clinical and multimodal biomarker changes of Alzheimer disease in adults with Down syndrome	JAMA Neurol	Adjusted for sex (covariate)	Tau/Aβ	PET/MRI
Lao et al. (2020)	138 DS adults (age 50 ± 7) (54 F/84 M)	Alzheimer-Related Cerebrovascular Disease in Down Syndrome	Ann Neurol	APOE4 carrier men showed greater white matter hyperintensity volume compared to women; no differences observed in striatal amyloid	Aβ	PET/MRI
Tudorascu et al. (2020)	135 DS adults, 21 non-DS adults (age 39.05 ± 8.4) (80 F/76 M)	Relationship of amyloid beta and neurofibrillary tau deposition in Neurodegeneration in Aging Down Syndrome (NiAD) study at baseline	Alzheimer Dement (NY)	Adjusted for sex (covariate)	Tau/Aβ	PET/MRI
Cody et al. (2020)	47 DS adults (age 26–56) (24 F/23 M)	Association of Sleep with Cognition and β-Amyloid Accumulation in Adults with Down Syndrome	Neurobiol Aging	No sex comparison performed	Aβ	PET/MRI
Keator et al. (2020)	79 DS adults (age 40–64) (26 F/53 M)	Down syndrome: Distribution of brain amyloid in mild cognitive impairment	Alzheimers Dement (Amst)	Small sample size of females, unable to evaluate sex differences	Aβ	PET/MRI
Hartley et al. (2020)	118 DS adults (age 37.24 ± 7.70) (61 F/57 M)	Cognitive indicators of transition to preclinical and prodromal stages of Alzheimer's disease in Down syndrome	Alzheimers Dement (Amst)	Rate of cognitive decline did not differ by biological sex	Aβ	PET/MRI
Cole et al. (2017)	46 DS adults (age 28–65) (21 F/25 M)	Brain-predicted age in Down syndrome is associated with beta amyloid deposition and cognitive decline	Neurobiol Aging	No association between sex and PiB status	Aβ	PET/MRI
Lao et al. (2016)	72 DS adults (age 30–53) (34 F/38 M)	The effects of normal aging on amyloid-β deposition in non-demented adults with Down syndrome as imaged by [11C]PiB	Alzheimers Dement	No significant differences in Aβ deposition observed between sexes	Aβ	PET/MRI
Handen et al. (2012)	8 DS adults (age 20–44) (2 F/6 M)	Imaging brain amyloid in non-demented young adults with Down syndrome using Pittsburgh compound B	Alzheimers Dement	Underpowered for sex comparison	Aβ	PET/MRI
Landt et al. (2011)	9 DS adults (age 25–64) (4 F/5 M)	Using positron emission tomography and carbon 11–labeled pittsburgh compound B to image brain fibrillar β-amyloid in adults with Down syndrome	JAMA Neurology	Underpowered for sex comparison	Aβ	PET/MRI

### Sex differences in people with DS observed by neuroimaging studies—PET

Positron emission topography (PET) imaging can reveal the location of key pathologies *in vivo* across the lifespan. PET studies identify differences in AD pathology using ligands that bind to Aβ and tau. In LOAD, although most studies report no sex differences seen through amyloid-PET burden, adjusting for amyloid levels reveals greater tau burden in the entorhinal cortex in women compared to men (Buckley et al., 2019b). However, despite similar levels of amyloid by PET, women perform better overall in cognitive testing exams (Sperling et al., 2020). Buckley et al. (2021) found post-menopausal women had higher tau-PET burden in the lateral occipital in comparison to pre-menopausal women and age-matched men. Given these observed sex differences in the neurotypical population, this begs the question of whether the same is seen in DS.

In DS, amyloid and tau PET imaging reveal many key AD signatures common to LOAD and to autosomal dominant AD with other interesting differences though few have examined sex differences directly (Head et al., 2018; Neale et al., 2018) (Table 2). In a study focused on nine individuals with DS, adults with DS over the age of 45 years had significant amyloid deposition in regions associated with AD compared to controls (Landt et al., 2011) including the inferior parietal, lateral occipital, and superior frontal regions (Keator et al., 2020). Interestingly, as more cohorts have been examined, the striatum is consistently identified as an area that is affected early by amyloid in DS (Handen et al., 2012; Cody et al., 2020) but whether sex differences contribute to this unique signature in DS has yet to be determined. In a cohort of 68 non-demented adults with DS (30–53 years), a comparison of males and females indicated no difference in the amount of amyloid by PET or regions affected (Lao et al., 2016), as has been corroborated in other studies (Cole et al., 2017).

We can hypothesize, however, that if aging in DS continues to show similarities to the neurotypical population, it is likely that sex differences would be seen in tau deposition relative to levels of amyloid. There are fewer tau-PET studies in aging cohorts of people with DS, but several studies now confirm that tau binding does not occur until after amyloid is present (Rafii et al., 2017; Tudorascu et al., 2020; Zammit et al., 2021). Further, tau-PET demonstrates accumulation of tau consistent with Braak staging used in neuropathology studies (Rafii et al., 2017; Tudorascu et al., 2020; Zammit et al., 2021). To date, no studies have examined tau sex differences in DS, which will be important to observe in future studies and particularly whether similar differences in tau levels in females as described by (Buckley et al., 2019b, 2021) can be identified in DS.

Though a comparison of amyloid or tau-PET imaging data in pre and post-menopausal women with DS has not been described, it is likely there would be differences given that earlier age of onset in DS is associated with increased risk of AD, it is likely there would be differences observed in PET imaging as well. Sex differences in the general population highlight the need for a similar focus when studying aging and AD pathogenesis in people with DS using MRI/PET imaging outcomes in people with DS. However, many PET studies in people with DS adjust for sex as a covariate, rather than investigating it as a biological variable, which opens many exciting opportunities for future research.

### Sex differences in DS observed in fluid biomarker studies

While imaging techniques allow us a window into the brain, fluid biomarkers provide additional non-invasive outcome measures. In LOAD, common fluid biomarkers include levels of tau, phosphorylated tau and Aβ, but also neurofilament light (NfL), corticotrophin releasing factor (CRF), and many others (Ferretti et al., 2018; Mielke, 2020). No significant sex differences in levels of Aβ in cerebrospinal fluid (CSF) have been reported in LOAD cohorts, which aligns with findings in amyloid-PET (Li et al., 2017; Ferretti et al., 2018; Bouter et al., 2019; Buckley et al., 2019a). Similarly in studies of plasma tau or phosphorylated tau levels in CSF or plasma in the LOAD population suggest no differences between males and females (Li et al., 2017; Ferretti et al., 2018; Bouter et al., 2019; Mielke et al., 2019). However, several studies report women who have lower Aβ in the CSF may be at higher risk for accumulating phosphorylated tau in comparison to men (Koran et al., 2017; Buckley et al., 2019a). Neurofilament light (NfL) is a cytoplasmic protein found in neurons which, if detected at the periphery, indicates axonal damage in the brain (Gaetani et al., 2019). Several studies report that men have higher NfL levels in CSF (Mattsson et al., 2016; Bridel et al., 2019; Mielke et al., 2019), though plasma and serum studies have reported no such differences (Mattsson et al., 2016; Chatterjee et al., 2018).

Many key fluid biomarkers of AD are now being investigated as biomarkers in DS, such as Aβ, tau, markers of inflammation and neuronal damage (Table 3). Elevated levels of plasma Aβ40 and Aβ42 are documented in DS, and elevated levels are correlated to development of dementia (Alhajraf et al., 2019). In a study of 62 individuals with DS (37 females, 25 males), plasma levels of total tau increased in women with DS (both AD and mild cognitive impairment groups) compared to controls, whereas this difference did not translate to men (Dang et al., 2021). A possible mechanism for increased tau pathology in females could be due to elevated levels of CRF, compared to males, which is associated with elevated phosphorylated tau levels (Bangasser et al., 2017). One study in 236 people with DS observed a 14.8% difference in plasma NfL in men with DS compared to women with DS (>35 years) at baseline (Carmona-Iragui et al., 2021). Carmona-Iragui et al. (2021) also found a 3.8% increase in NfL plasma levels with each year from baseline, though from this point on sex was investigated as a covariate and not as a biological variable. The discrepancy between men and women at baseline suggests women with DS may present with neuronal damage earlier than their male counterparts. It would be interesting to compare pre and post-menopausal women with DS in relation to NfL levels to determine if NfL drops with menopause.

**Table 3:** Literature on sex differences in Alzheimer's disease biomarkers in Down syndrome.

**References**	**Sample** **(age in years)** **(female/male)**	**Title**	**Source**	**Findings**
Pentz et al. (2021)	36 DS adults; 16 non-DS adults (non-AD DS: 39.9 ± 2.9; DSAD: 52.9 ± 2.9; non-DS: 52.8 ± 1.1) (25 F/27 M)	Nerve growth factor (NGF) pathway biomarkers in Down syndrome prior to and after the onset of clinical Alzheimer's disease: a paired CSF and plasma study	Alzheimers Dement	Males had higher levels of CSF neuroserpin in non-trisomic and DSAD, but females had higher levels in AD-asymptomatic DS; males had higher levels MMP-9 in plasma and higher levels of MMP-3 in plasma and CSF across all groups
Carmona-Iragui et al. (2021)	236 DS adults (age ≥ 18) (75 F/90 M)	Diagnostic and prognostic performance and longitudinal changes in plasma neurofilament light chain concentrations in adults with Down syndrome: a cohort study	Lancet Neurol	Males with DS showed 14.8% lower concentrations of plasma NfL than females with DS
Petersen et al. (2021)	305 DS adults (age ≥ 25) (113 F/135 M)	Plasma total-tau and neurofilament light chain as diagnostic biomarkers of Alzheimer's disease dementia and mild cognitive impairment in adults with Down syndrome	J Alzheimers Dis	Addition of sex improved accuracy of model
Dang et al. (2021)	275 DS adults (169 F/106 M)	Sex differences in levels of plasma neurofilament light and total tau in adults with Down syndrome	Alzheimers Dement	Women with DS had higher levels of total tau compared to controls; difference not seen in men
Petersen et al. (2020)	305 DS individuals (age cog. stable: 42.4 ± 9.1; MCI: 52.9 ± 6.9; AD: 54.3 ± 6.2) (153 F/183 M)	Proteomic profiles for Alzheimer's disease and mild cognitive impairment among adults with Down syndrome spanning serum and plasma: an Alzheimer's Biomarker Consortium–Down Syndrome (ABC–DS) study	Alzheimers Dement (Amst)	Addition of sex improved accuracy of model to aid in diagnosis of early cognitive decline
Hamlett et al. (2017)	47 DS individuals; 37 non-DS individuals (age DS: 8–62; non-DS: 8–77) (41 F/43 M)	Neuronal exosomes reveal Alzheimer's disease biomarkers in Down syndrome	Alzheimers Dement	Men with DS (age > 35) had 34% higher exosomal P-T181-Tau compared to age-matched women with DS; opposite effect seen in non-trisomic group. No sex differences observed in exosomal Aβ1-42 and P-S396-Tau in both trisomic and non-trisomic groups.

Other emerging biomarkers in AD relate to the nerve growth factor (NGF) pathway, which is responsible for preserving cholinergic activity (Capsoni and Cattaneo, 2006). An investigation into the NGF metabolism pathway reveals several key differences between men and women with DS. Notably, in a neurotypical control group and in people with DS with AD groups, men had overall higher levels of neuroserpin, a protein belonging to the serine protease inhibitor family and elevated in AD (Subhadra et al., 2013; Pentz et al., 2021). In DS without AD, women had elevated levels of neuroserpin in CSF. In other markers MMP-3 and MMP-9, however, men had higher levels compared to women across all status groups (Pentz et al., 2021). Further, one of the goals of the fluid biomarkers field is to develop predictive models with enough sensitivity and accuracy to diagnose a person with early cognitive decline. Multiple studies have found accuracy improves when sex is included as a metric in these models (Petersen et al., 2020, 2021).

The study of extracellular vesicles or exosomes, from plasma provides a unique window into possible protein changes in the brain. One study examining exosomal biomarkers in DS found that men with DS over the age of 35 had 34% higher levels of P-T181-Tau compared to age-matched women with DS. Notably, the exact opposite effect was observed in a control group of age-matched participants, with the females having 34% higher levels of P-T181-Tau compared to men (Hamlett et al., 2017, 2018). These differences in AD markers, though sometimes subtle, may be due in part to hormone involvement, which will be explored in the next section.

## Sex differences in AD neuropathology in DS

Attempts to characterize sex differences in neuropathology in LOAD are few in number, though some studies provide consistent results highlighting women are affected at higher rates than men (Oveisgharan et al., 2018). Oveisgharan et al. (2018) found that among an autopsy cohort of 718 individuals, women were 35% more likely to have AD by pathological diagnosis. There is also likely an age-related interaction on sex differences as one study observed women over the age of 80 were more likely to have AD pathology than men (Nelson et al., 2010). Additionally, female APOE4 carriers exhibit higher plaque density than male carriers, suggesting an interaction between APOE status and sex (Corder et al., 2004).

Studies of sex effects on the extent of plaque or neurofibrillary tangle pathology at autopsy in DS brain doners are still limited primarily due to the lack of availability of sufficient case numbers (Table 1). An early study by Raghavan et al. (1994) examined neuropathology in the postmortem brains of 28 individuals with DS. In their study, comprised of 17 males and 11 females, women with DS had significantly higher mean tau neurofibrillary tangle burden compared to males. Of those with AD pathology present, all females had tau neurofibrillary tangle counts above 10 per mm^2^, whereas nearly half of the males had absent or low tau neurofibrillary tangle burden. When assessing amyloid plaques (SP), however, they found no significant differences between sexes (Raghavan et al., 1994). Further, the same subset of men also presented with no clinical dementia along with the lower tangle burden, despite having high SP loads (Raghavan et al., 1994). These further underscore tau as a key marker for sex interactions in DSAD, which should be investigated in future studies.

## Potential mechanisms driving sex differences in Alzheimer's disease in down syndrome

### The influence of hormones and menopause

Sex differences in biological aging may be driven through changes in hormone levels and in women, linked to menopause. Hormones exert many functions throughout the body and brain. In determining secondary sex characteristics, estrogen and testosterone are the two hormones most often implicated. Estrogen can exist in multiple biologically active forms, the main one being estradiol. However, aside from estrogen and testosterone, there are several other hormones that are dynamically and differentially regulated in men and women. The correlation between brain function and hormones is not a new concept, and cycles of cognitive patterns as a result of sex hormone fluctuations in women vs. men were previously reported (Kimura and Hampson, 1994).

Menopause, characterized by a large drop in estrogen levels, is a significant hormonal event affecting many systems. Women with DS experience menopause, on average, 5–7 years earlier than the general population (Coppus et al., 2010; Zigman, 2013) and as mentioned previously, women with less bioavailable estrogen are at higher risk of dementia (Schupf et al., 2006). Estrogens act on two main receptors: estrogen receptor alpha (ERα) and estrogen receptor beta (ERβ) (Simpkins et al., 2012). There is a third type, the G-protein coupled receptor (GPER), which was more recently discovered (Simpkins et al., 2012). All three estrogen receptors are expressed throughout the brain, with higher levels present in the frontal cortex and hippocampus (Almey et al., 2015). Whether the functions of ERα and ERβ confer neuroprotection is still under investigation. Some studies show ERα is more neuroprotective than ERβ, while others have shown the opposite (Simpkins et al., 2012; Baumgartner and Daniel, 2021; Ishunina, 2021). How these receptors change throughout the life course in the brain in response to changing hormone levels also remains to be elucidated.

**Figure 1:**
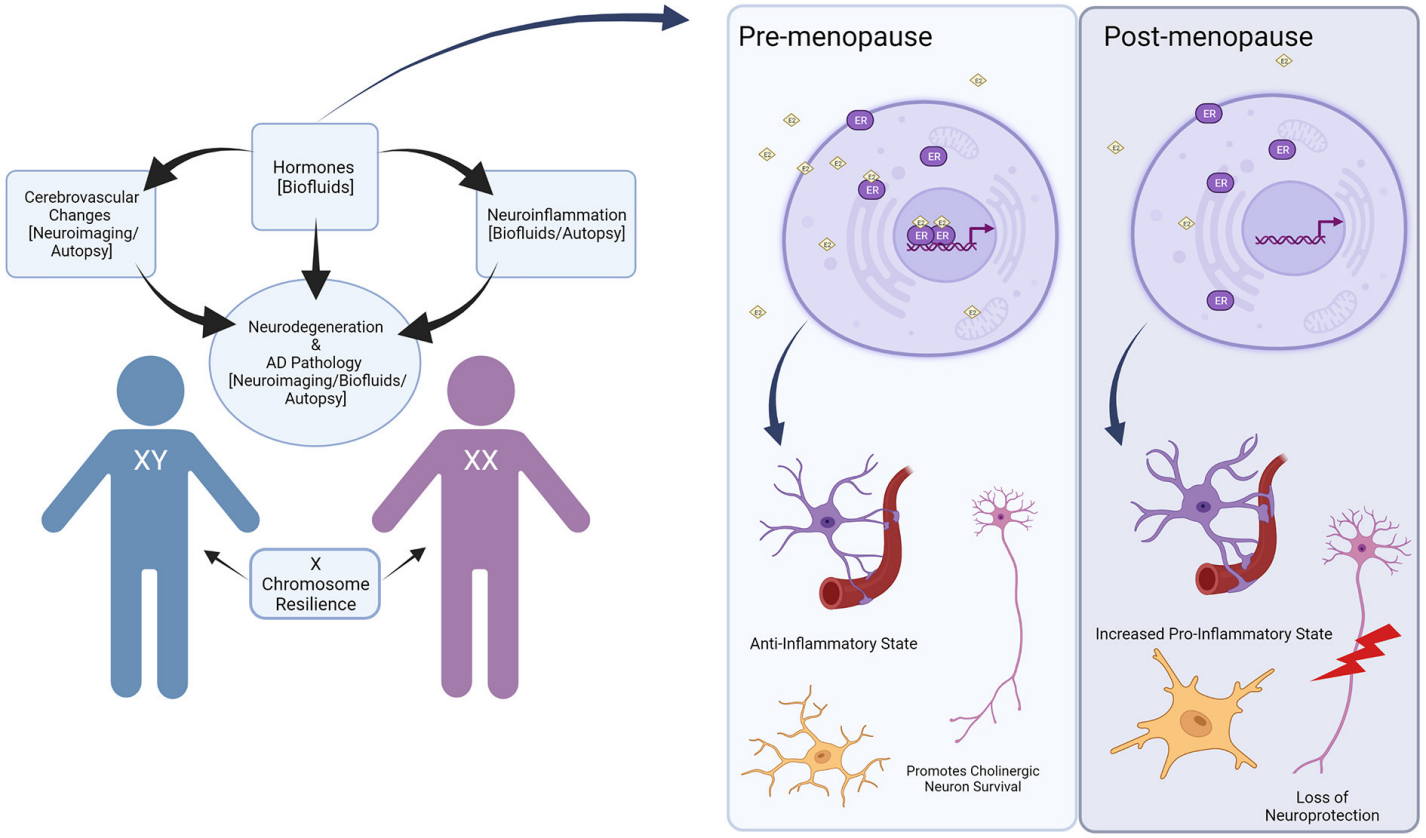
Overview of sex differences in Down syndrome and Alzheimer's disease. Hormones can contribute to cerebrovascular changes and neuroinflammation in the brain, which can lead to increased Alzheimer's disease pathology. These changes can be identified through neuroimaging, biofluids, and autopsy studies, though only a handful have investigated sex differences directly. Hormones such as estrogen can have a particularly beneficial effect, which is then withdrawn during hormonal events such as menopause. In the pre-menopausal state, bioavailable estrogen (represented as E2) acts on estrogen receptors (ER) to promote an anti-inflammatory state in the brain while maintaining neuronal health. In the post-menopausal state, lower levels of estrogen lead to increased pro-inflammatory states and loss of neuroprotection. In addition to hormonal effects, the X chromosome may contribute to resilience in aging and improve cognitive outcomes, though this has not been studied in the context of DS. Created with BioRender.com.

Given estrogen's many neuroprotective effects, it is likely that menopause contributes to adverse effects on cognition and an increased risk of AD in women (Brinton, 2001; Garcia-Segura et al., 2001; Andrew and Tierney, 2018) (Figure 1). In the general population, the neuroprotective properties of estrogen are related to reduced Aβ toxicity in the brain though exact mechanisms are unclear, growth and survival of cholinergic neurons, and anti-inflammatory effects in the brain have been suggested (Spampinato et al., 2012). Several studies show estrogen promotes metabolism of toxic Aβ, while other hormones, such as corticosterone, may exacerbate pathologic Aβ deposition (Jaffe et al., 1994; Goodman et al., 1996; Xu et al., 2006). Interestingly, estrogen can also have effects independent of receptor activity as well and appear to act on mitochondria by stabilizing membrane potentials and reducing depletion of adenosine triphosphate (ATP) (McEwen, 2002). In AD, estrogen promotes the metabolism of APP and protects against the toxicity of Aβ accumulation, which we will discuss later in this review.

Estrogen is also closely related to testosterone, with the latter being converted to the former through enzymatic activity of aromatase. Older men have higher levels of circulating estrogen when compared to post-menopausal women (Baker Frost et al., 2019). Given that men do not experience a similar drop in testosterone as women do with estrogen, their levels remain constant throughout their life. Similar to estrogen, lower levels of testosterone in plasma are associated with higher risk of sporadic AD in elderly men (Lv et al., 2016), suggesting the neuroprotective role sex hormones possess. Additionally, gonadotrophin releasing hormone (GnRH), which acts on the pituitary gland to stimulate hormone release, is associated with an increase in levels of estradiol in the hippocampus, resulting in increased pyramidal neuron activity and improved memory outcomes (Marbouti et al., 2020). This suggests a potential cascade of hormones that may be involved in memory preservation and could become less effective following hormonal changes such as menopause. Hormones could contribute to the oftentimes conflicting information on the risk of AD in women with DS, and may represent an explanatory factor for often contradictory outcomes from HRT trials for AD.

In women with DS, there is a strong association of lower levels of bioavailable estrogen and higher risk of AD reported in a longitudinal research cohort study (Schupf et al., 2006, 2018). Interestingly, several sex differences in mouse models of DS have been described. In a behavioral phenotype study, environmental enrichment led to improved spatial memory and acquisition scores in female Ts65Dn mice compared to males, suggesting that sex may have a modulatory role in cognition (Martínez-Cué et al., 2002). These differences may be due, to some extent, to circulating hormones in response to stimulation. Another study found that female Ts65Dn mice have lower adrenocorticotropic hormone at rest compared to males, but elevated levels of corticosterone under stress conditions (Martínez-Cué et al., 2006). Whether there are other hormonal differences in the Ts65Dn mouse model which may be related to aging remains unstudied but could pose a potential route of investigation in the future.

### Sex differences in cerebrovascular pathology

Sex differences may also impact the onset or extent of cerebrovascular pathology. The brain is comprised of an intricate network of vasculature supplying cells with oxygen and other essential nutrients. The blood brain barrier (BBB) separates the central nervous system, bathed in cerebral spinal fluid (CSF), from circulatory blood. It is a complex junction composed of several cells working in harmony to filter necessary components into the brain, while keeping harmful components out. The main components are comprised of capillary endothelial cells, the basement membrane, and astrocyte end feet (Dotiwala et al., 2021). Other cells, such as pericytes, are also at work. In neurodegenerative conditions, the BBB can weaken and allow the flow of neurotoxic substances into the brain (Kalaria, 1999), and several studies have linked BBB breakdown to cognitive decline (Bowman et al., 2018; Nation et al., 2019; Montagne et al., 2020; Li et al., 2021). Increasing levels of S100 calcium-binding protein (S100B), secreted from astrocytes may indicate a breakdown of the BBB (Griffin et al., 1989) and S100B is upregulated in females (Weis et al., 2021).

There are several significant differences in both systemic and central vascular function in people with DS. Differences in cerebrovascular pathology in people with DS could be driven by systemic congenital cardiac factors that are associated with trisomy 21 (Mito et al., 1991; Wilcock et al., 2016). For example, female infants with DS have an increased rate of heart defects compared to males. Compared to the general population, this difference in heart defects is significant (Diogenes et al., 2017; Santoro et al., 2018), begging many questions regarding what peripheral vascular differences in DS could also impact the development of AD. Interestingly, though the development of vascular pathology is more likely, people with DS tend to have lower blood pressure and less frequent atherosclerosis, which may aid in preserving vascular function later in life (Wilcock et al., 2016).

Cerebrovascular pathology is more extensive with increasing age in people with DS compared to the neurotypical population. People with DS accumulate cerebrovascular pathology at a higher frequency compared to sporadic AD, typically in the form of cerebral amyloid angiopathy (CAA) (Head et al., 2017). This is likely due to increased expression of APP and associated overproduction of Aβ. In addition to CAA, people with DS may also develop microbleeds (MBs), which could indicate breakdown of the blood brain barrier. One study published in 2019 found that there was an increase in MBs along with higher levels of CAA and both increased with age (Helman et al., 2019).

Given the extensive CAA reported in the brains of people with DS, it is likely that the BBB may play a role in age-related cognitive decline in DS and may be differentially affected by sex. S100 calcium-binding protein (S100B) associated with astrocytes critical for BBB integrity is elevated in DS and AD (Griffin et al., 1989) although this may be due, in part, to the S100B gene being on chromosome 21. Estrogen may protect the integrity of the BBB by improving tight junction functionality in the endothelial cell layer (Maggioli et al., 2016). One could speculate that if estrogen does exert protective effects on the BBB, then post-menopausal women, who experience a drastic drop in estrogen, would be at an even greater risk for neurodegeneration due to BBB breakdown. How and when cerebrovascular pathology and BBB integrity may be affected differentially in men and women with DS will benefit from more research. As mentioned previously, evidence from MRI studies suggest higher WMH burden in men with DS compared to women, suggesting sex differences in cerebrovascular pathology may be an important modifier of age of onset of dementia and represents an opportunity for future research.

### Immune system involvement and neuroinflammation

Cerebrovascular pathology and loss of BBB integrity with aging and AD both in the general population and in DS can drive inflammation through the leakage of serum proteins into the brain. The neuroimmune system changes dynamically with age and it is one of the key drivers in the progression of AD in the general population (Cao and Zheng, 2018; Newcombe et al., 2018). From a broader perspective, neurotypical women show more robust immune responses and greater resistance to infection (Gaillard and Spinedi, 1998). Consequently, the instance of autoimmune diseases is greater in women (Whitacre, 2001). These differences suggest varied mechanistic underpinnings driving immune development in men and women. In the brain, microglia, the innate immune cells in the central nervous system (CNS), display their own unique molecular sex differences in the neurotypical population (Lenz and McCarthy, 2014). In addition to microglia, astrocytes are a highly abundant non-neuronal cell type in the CNS. They are critically involved in important processes such as synapse formation, neurovascular coupling, communication and support to neurons, and play a significant role in AD development (for a complete review please see references Matias et al., 2019; Han et al., 2021). However, sex differences in astrocytes in DS have not been studied systematically in the neurotypical population. As mentioned in the previous section, ERs are associated with regulating inflammation, specifically in terms of reducing amyloid-induced inflammation, and they are expressed in astrocytes as well as microglia (Crespo-Castrillo and Arevalo, 2020) (Figure 1).

Peripheral immune system dysfunction in people with DS includes the frequent presentation of autoimmune disorders (Guaraldi et al., 2017). Though to date no studies have specifically investigated sex differences frequencies of DS autoimmune disorders, hypothyroidism appears to be equivalent in prevalence between men and women with DS (Popova et al., 2008), which differs from the general population. However, studies investigating sex differences in the immune system of people with DS are few in number. Within the DS population, a unique phenotype of inflammation is observed (Rodrigues et al., 2014; Wilcock et al., 2015). Females with DS also showed an increase in interleukin-6 and interleukin-8 levels when compared to males (Flores-Aguilar et al., 2020).

Neuroinflammation is particularly important in the development and progression of AD, which holds true in the DS population as well. Understanding specific sex differences that may be related mechanistically to neuroinflammation in the DS population will help to better target future therapeutic treatments.

### Synapse proteins and function

Cerebrovascular pathology, neuroinflammation and the accumulation of Aβ and tau neurofibrillary tangles lead to synapse loss and neurodegeneration in AD. Under normal conditions, synapses are dynamically regulated and responsible for intact cognitive performance. LOAD is associated with a significant loss of synapses and synapse proteins such as synaptophysin, SNAP-25 and PSD-95 (DeKosky and Scheff, 1990; Terry et al., 1991; Rajendran and Paolicelli, 2018). SNAP-25 is an important component of the SNARE complex, which aids in vesicle trafficking. Synaptophysin, on the other hand, is a protein found in synaptic vesicles (Cousin, 2021). In sporadic AD, researchers have noted recently that one glutamatergic receptor, the metabotropic glutamate receptor 5 (mGluR5), may have different mechanisms of action in men and women. The mGluR5 receptor forms a complex with cellular prion protein (PrP^c^) and Aβ oligomers to promote pathologic amyloid accumulation in men, whereas this complex does not form in women (Abd-Elrahman et al., 2020).

In DS, synaptic development is significantly dysregulated, which likely also contributes to the AD phenotype development or enhanced vulnerability to AD pathology later in life (Engidawork and Lubec, 2001; El Hajj et al., 2016). Studies using human induced pluripotent stem cell (iPSC)-derived cortical neurons from people with DS that are injected into the brains of mice, leads to an overall lower network activity as well as lower rate of dendritic spine turnover (Real et al., 2018). In terms of sex differences in proteins associated with synaptic dysfunction, women across both DS and non-DS groups showed elevated levels of SNAP-25 in comparison to men. In contrast, synaptophysin protein levels are significantly increased in both neurotypical men and men with DS in comparison to women (Downes et al., 2008). The fact that there are opposing levels of SNAP-25 and synaptophysin in DS could highlight different mechanisms of synaptic dysfunction in males and females with age and AD in DS.

### The contribution of sex chromosomes

An emerging field of study suggests resilience in aging could stem from activation of sex chromosomes. Sex chromosomes, existing in pairs of XX or XY, can also be triplicated during development resulting in abnormal development and cognition such as in triple X syndrome. The impact of sex chromosomes in the context of neurodegeneration and aging is a rapidly evolving field (Snyder et al., 2016; Dubal, 2020), and available evidence points to sex chromosomes having a critical impact on cognition throughout the lifespan. Females have two X chromosomes in their genome, one of which is silenced during development. However, the pattern of silencing is not identical throughout tissues, with both maternal and paternal X chromosomes having an equal potential to be silenced (Deng et al., 2014). Females are characterized by a mosaic of cells with contributions from either their maternal or paternal X chromosome. Even after an X chromosome is silenced, some genes can still be expressed, known as gene escape, which can influence aging (Berletch and Disteche, 2012). The impact of gene escape from sex chromosomes on AD was investigated in a mouse model of AD by varying sex chromosome dosage. In mice with only one X chromosome (XY or XO), there is a higher mortality rate than in those with two X chromosomes (XX). In addition, a candidate gene, KDM6A, not silenced during X-inactivation, is associated with improved cognitive outcomes in human studies (Davis et al., 2020).

The X chromosome, is larger in size and contains more protein-coding genes than the Y chromosome, making it a prime target for genomic studies. According to a human genomics study, females exhibit a higher diversity of immune responses due to the random inactivation of one of the X-chromosomes. As mentioned previously, not all of the X-chromosome is silenced and varies across individuals. It is possible that later in life more genes escape the silenced X-chromosome, leading to more extensive immune responses, and, as a consequence, more neuroinflammation that contributes to neurodegeneration. The X chromosome is of particular interest to AD as it contains a few genes, such as MECP2, known to contribute to neurodegenerative vulnerability (Bajic et al., 2020). It is also important to note that although we refer to sex as a binary here, there are individuals with other variations such as XXY and XXX. Studies demonstrate some shared characteristics in executive functions between people with DS and people with trisomy of sex chromosomes (Lee et al., 2015).

Although there are fewer genes that encode for proteins on the Y chromosome, a deletion could significantly contribute to downstream effects. In addition, later in life, men can experience the loss of a Y chromosome (LOY). One study has shown that men with higher levels of LOY in blood cells also had greater risk of AD development (Dumanski et al., 2016). Some regions of the Y chromosome can be randomly deleted in men with DS, though the mechanism by which this occurs is unknown (Yasin et al., 2014). To date, sex chromosomes in DS have not been studied in the context of neurodegeneration, but it is likely that changes in these chromosomes could have significant impact in this population as people with DS are more likely to develop AD.

## Conclusions

Examining sex differences in DS research as to why these differences occur mechanistically and how they impact AD development and progression are opportunities for the future. In this review, we highlighted studies of sex differences in the current literature related to LOAD and when present, studies that included people with DS. Although the research is somewhat inconsistent in terms of outcomes, there are intriguing suggestions that estrogen may be a key player and that estrogen replacement therapy may be a consideration to improve healthy brain aging in women with DS (Schupf et al., 2003, 2006, 2018). However, to date, there are no clinical trials involving people with DS and hormone therapy for the purpose of improving cognition. In sporadic AD, clinical trials are inconclusive in terms of whether these therapies are beneficial. Some trials show modest cognitive improvement, whereas others observed no change or even a decline in cognition in women administered HRT (Whitmer et al., 2011).

If estrogen has neuroprotective properties, why would clinical trials produce mixed results? The answer may not be straightforward. During menopause, there are several hormonal fluctuations that occur alongside a massive drop in estrogen levels. Timing of HRT may be critical to its benefit or adverse impact. Some studies also suggested that the peri-menopausal (transitional) period could be a pro-inflammatory state setting the stage for neurodegenerative-related inflammation (McCarthy and Raval, 2020). This is in agreement with findings in LOAD, as one study noted women who had undergone surgical menopause at younger ages developed AD pathology and cognitive decline faster than those who had undergone the procedure later in life (Bove et al., 2014). Since women with DS experience menopause earlier than the neurotypical population, this could put them at especially high risk for inflammation early relative to their male counterparts (Figure 1).

It is also worth noting that not all clinical trials focused on estrogen use similar approaches. Some have administered hormone replacement therapy, which often involved a combination of hormones, while others have administered estrogen replacement therapy. Another question that remains is the mechanism of action: are the effects observed a consequence of the hormones themselves, or are they due to receptor function? Estrogen receptor agonists, which selectively mimic the effects of estrogen can also lower inflammation (Chakrabarti et al., 2014).

In the future, it will be crucial to continue investigating sex differences in aging and AD pathogenesis in DS and the underlying mechanisms, especially as the lifespan among people with DS increases with improved healthcare and lifestyle. Studies will benefit from the inclusion of an equally distributed representation of males and females as participants and consider and analyze the specific contributions of sex differences to cognition and AD pathogenesis in analyses. Future intervention studies may benefit from targets that account for sex differences that in turn, lead to health benefits for people with DS.

## Author contributions

Conception, design of review, and manuscript writing and editing: EA, AM, and EH. Supervision: EH. All authors contributed to the article and approved the submitted version.

## Funding

This study was supported by grants from the National Institute of Health (NIH) (NIH/NIA U19AG068054, P50AG16573, and P30AG066519), the Brightfocus Foundation Grant BFF170008, and the UCI Alzheimer's Disease Research Center/Women's Alzheimer's Movement (UCI MIND/WAM 01-2021) Pilot Grant.

## Conflict of interest

The authors declare that the research was conducted in the absence of any commercial or financial relationships that could be construed as a potential conflict of interest.

## Publisher's note

All claims expressed in this article are solely those of the authors and do not necessarily represent those of their affiliated organizations, or those of the publisher, the editors and the reviewers. Any product that may be evaluated in this article, or claim that may be made by its manufacturer, is not guaranteed or endorsed by the publisher.

## References

[B1] Abd-Elrahman K. S. Albaker A. de Souza J. M. Ribeiro F. M. Schlossmacher M. G. Tiberi M.. (2020). Aβ oligomers induce pathophysiological mGluR5 signaling in Alzheimer's disease model mice in a sex-selective manner. Sci. Signal. 13, 494. 10.1126/scisignal.abd2494 33323410

[B2] Alhajraf F. Ness D. Hye A. Strydom A. (2019). Plasma amyloid and tau as dementia biomarkers in Down syndrome: systematic review and meta-analyses. Dev. Neurobiol. 79, 684–698. 10.1002/dneu.22715 31389176 PMC6790908

[B3] Almey A. Milner T. A. Brake W. G. (2015). Estrogen receptors in the central nervous system and their implication for dopamine-dependent cognition in females. Horm. Behav. 74, 125–138. 10.1016/j.yhbeh.2015.06.010 26122294 PMC4820286

[B4] Altmann A. Tian L. Henderson V. W. Greicius M. D. (2014). Sex modifies the APOE-related risk of developing Alzheimer's disease. Ann. Neurol. 75, 563. 10.1002/ana.24135 24623176 PMC4117990

[B5] Andrew M. K. Tierney M. C. (2018). The puzzle of sex, gender and Alzheimer's disease: why are women more often affected than men? Women's Heal. 14, 1745506518817995. 10.1177/1745506518817995

[B6] Annus T. Wilson L. R. Acosta-Cabronero J. Cardenas-Blanco A. Hong Y. T. Fryer T. D.. (2017). The Down syndrome brain in the presence and absence of fibrillar β-amyloidosis. Neurobiol. Aging 53, 11–19. 10.1016/j.neurobiolaging.2017.01.009 28192686 PMC5391869

[B7] Antonarakis S. E. Skotko B. G. Rafii M. S. Strydom A. Pape S. E. Bianchi D. W.. (2020). Down syndrome. Nat. Rev. Dis. Prim. 6, 1–20. 10.1038/s41572-019-0143-7 32029743 PMC8428796

[B8] Bajic V. P. Essack M. Zivkovic L. Stewart A. Zafirovic S. Bajic V. B.. (2020). The X files: “the mystery of X chromosome instability in Alzheimer's disease.” Front. Genet. 10, 1368. 10.3389/fgene.2019.01368 32047510 PMC6997486

[B9] Baker Frost D. Wolf B. Peoples C. Fike J. Silver K. Laffoon M.. (2019). Estradiol levels are elevated in older men with diffuse cutaneous SSc and are associated with decreased survival. Arthritis Res. Ther. 21, 85. 10.1186/s13075-019-1870-6 30940202 PMC6444502

[B10] Bangasser D. A. Dong H. Carroll J. Plona Z. Ding H. Rodriguez L.. (2017). Corticotropin-releasing factor overexpression gives rise to sex differences in Alzheimer's disease-related signaling. Mol. Psychiatry 22, 1126. 10.1038/mp.2016.185 27752081 PMC5395355

[B11] Baumgartner N. E. Daniel J. M. (2021). Estrogen receptor α: a critical role in successful female cognitive aging. Climacteric 24, 333–339. 10.1080/13697137.2021.1875426 33522313 PMC8273070

[B12] Bejanin A. Iulita M. F. Vilaplana E. Carmona-Iragui M. Benejam B. Videla L.. (2021). Association of apolipoprotein E ε4 allele with clinical and multimodal biomarker changes of alzheimer disease in adults with down syndrome. JAMA Neurol. 78, 937–947. 10.1001/jamaneurol.2021.1893 34228042 PMC8261691

[B13] Benejam B. Videla L. Vilaplana E. Barroeta I. Carmona-Iragui M. Altuna M.. (2020). Diagnosis of prodromal and Alzheimer's disease dementia in adults with Down syndrome using neuropsychological tests. Alzheimers Dement 12, e12047–e12047. 10.1002/dad2.12047 PMC7322242 32613076

[B14] Berletch J. B. Disteche C. M. (2012). Genes the escape X Inactivation. Hum. Genet. 130, 237–245. 10.1007/s00439-011-1011-z PMC3136209 21614513

[B15] Bouter C. Vogelgsang J. Wiltfang J. (2019). Comparison between amyloid-PET and CSF amyloid-β biomarkers in a clinical cohort with memory deficits. Clin. Chim. Acta 492, 62–68. 10.1016/j.cca.2019.02.005 30735665

[B16] Bove R. Secor E. Chibnik L. B. Barnes L. L. Schneider J. A. Bennett D. A.. (2014). Age at surgical menopause influences cognitive decline and Alzheimer pathology in older women. Neurology 82, 222. 10.1212/WNL.0000000000000033 24336141 PMC3902759

[B17] Bowman G. L. Dayon L. Kirkland R. Wojcik J. Peyratout G. Severin I. C.. (2018). Blood-brain barrier breakdown, neuroinflammation, and cognitive decline in older adults. Alzheimer's Dement. 14, 1640–1650. 10.1016/j.jalz.2018.06.2857 30120040

[B18] Bridel C. van Wieringen W. N. Zetterberg H. Tijms B. M. Teunissen C. E. the NFL Group. (2019). Diagnostic value of cerebrospinal fluid neurofilament light protein in neurology: a systematic review and meta-analysis. JAMA Neurol. 76, 1035–1048. 10.1001/jamaneurol.2019.1534 31206160 PMC6580449

[B19] Brinton R. D. (2001). Cellular and molecular mechanisms of estrogen regulation of memory function and neuroprotection against Alzheimer's disease: recent insights and remaining challenges. Learn. Mem. 8, 121–133. 10.1101/lm.39601 11390632

[B20] Buckley R. F. Mormino E. C. Chhatwal J. Schultz A. P. Rabin J. S. Rentz D. M.. (2019a). Associations between baseline amyloid, sex and APOE on subsequent tau accumulation in cerebrospinal fluid. Neurobiol. Aging 78, 178. 10.1016/j.neurobiolaging.2019.02.019 30947113 PMC6545139

[B21] Buckley R. F. Mormino E. C. Rabin J. S. Hohman T. J. Landau S. Hanseeuw B. J.. (2019b). Sex differences in the association of global amyloid and regional tau deposition measured by positron emission tomography in clinically normal older adults. JAMA Neurol. 76, 542–551. 10.1001/jamaneurol.2018.4693 30715078 PMC6515599

[B22] Buckley R. F. O'Donnell A. McGrath E. R. Jacobs H. I. L. Satizabal C. L. Ghosh S.. (2021). Menopause moderates sex differences in tau PET signal: findings from the Framingham Study. Alzheimer's Dement. 17, 3–5. 10.1002/alz.054966

[B23] Burke S. L. Hu T. Fava N. M. Li T. Rodriguez M. J. Schuldiner K. L.. (2019). Sex differences in the development of mild cognitive impairment and probable Alzheimer's disease as predicted by hippocampal volume or white matter hyperintensities. J. Women Aging 31, 140–164. 10.1080/08952841.2018.1419476 29319430 PMC6039284

[B24] Cao W. Zheng H. (2018). Peripheral immune system in aging and Alzheimer's disease. Mol. Neurodegener. 13, 51. 10.1186/s13024-018-0284-2 30285785 PMC6169078

[B25] Capsoni S. Cattaneo A. (2006). On the molecular basis linking nerve growth factor (NGF) to Alzheimer's disease. Cell. Mol. Neurobiol. 26, 617–631. 10.1007/s10571-006-9112-2 PMC11520721 16944323

[B26] Carmona-Iragui M. Alcolea D. Barroeta I. Videla L. Muñoz L. Van Pelt K. L.. (2021). Diagnostic and prognostic performance and longitudinal changes in plasma neurofilament light chain concentrations in adults with Down syndrome: a cohort study. Lancet Neurol. 20, 605–614. 10.1016/S1474-4422(21)00129-0 34302785 PMC8852333

[B27] Chakrabarti M. Haque A. Banik N. L. Nagarkatti P. Nagarkatti M. Ray S. K. (2014). Estrogen receptor agonists for attenuation of neuroinflammation and neurodegeneration. Brain Res. Bull. 109, 22. 10.1016/j.brainresbull.2014.09.004 25245209 PMC4378838

[B28] Chatterjee P. Goozee K. Sohrabi H. R. Shen K. Shah T. Asih P. R.. (2018). Association of plasma neurofilament light chain with neocortical amyloid-β load and cognitive performance in cognitively normal elderly participants. J. Alzheimers. Dis. 63, 479–487. 10.3233/JAD-180025 29630554

[B29] Cody K. A. Piro-Gambetti B. Zammit M. D. Christian B. T. Handen B. L. Klunk W. E.. (2020). Association of sleep with cognition and beta amyloid accumulation in adults with Down syndrome. Neurobiol. Aging 93, 44–51. 10.1016/j.neurobiolaging.2020.04.018 32447011 PMC7380565

[B30] Cole J. H. Annus T. Wilson L. R. Remtulla R. Hong Y. T. Fryer T. D.. (2017). Brain-predicted age in Down syndrome is associated with beta amyloid deposition and cognitive decline. Neurobiol. Aging 56, 41–49. 10.1016/j.neurobiolaging.2017.04.006 28482213 PMC5476346

[B31] Coppus A. M. W. Evenhuis H. M. Verberne G. J. Visser F. E. Eikelenboom P. Van Gool W. A.. (2010). Early age at menopause is associated with increased risk of dementia and mortality in women with down syndrome. J. Alzheimer's Dis. 19, 545–550. 10.3233/JAD-2010-1247 20110600

[B32] Corder E. H. Ghebremedhin E. Taylor M. G. Thal D. R. Ohm T. G. Braak H. (2004). The biphasic relationship between regional brain senile plaque and neurofibrillary tangle distributions: modification by age, sex, and APOE polymorphism. Ann. N. Y. Acad. Sci. 1019, 24–28. 10.1196/annals.1297.005 15246987

[B33] Cousin M. A. (2021). Synaptophysin-dependent synaptobrevin-2 trafficking at the presynapse-Mechanism and function. J. Neurochem. 159, 78–89. 10.1111/jnc.15499 34468992

[B34] Crespo-Castrillo A. Arevalo M. A. (2020). Microglial and astrocytic function in physiological and pathological conditions: estrogenic modulation. Int. J. Mol. Sci. 21, 219. 10.3390/ijms21093219 PMC7247358 32370112

[B35] Crews L. Masliah E. (2010). Molecular mechanisms of neurodegeneration in Alzheimer's disease. Hum. Mol. Genet. 19, R12–R20. 10.1093/hmg/ddq160 20413653 PMC2875049

[B36] Dang L.-H. T. Krinsky-McHale S. J. O'Bryant S. Pang D. Zigman W. B. Silverman W.. (2021). Sex differences in levels of plasma neurofilament light and total tau in adults with Down syndrome. Alzheimer's Dement. 17, e055785. 10.1002/alz.055785

[B37] Davis E. J. Broestl L. Abdulai-Saiku S. Worden K. Bonham L. W. Miñones-Moyano E.. (2020). A Second X Chromosome Contributes to Resilience in a Mouse Model of Alzheimer's Disease. Available online at: https://www.science.org (accessed August 2, 2022). 10.1126/scitranslmed.aaz5677 PMC8409261 32848093

[B38] Dekker A. D. Ulgiati A. M. Groen H. Boxelaar V. A. Sacco S. Falquero S.. (2021). The behavioral and psychological symptoms of dementia in down syndrome scale (BPSD-DS II): optimization and further validation. J. Alzheimers. Dis. 81, 1505–1527. 10.3233/JAD-201427 33967040 PMC8293661

[B39] DeKosky S. T. Scheff S. W. (1990). Synapse loss in frontal cortex biopsies in Alzheimer's disease: correlation with cognitive severity. Ann. Neurol. 27, 457–464. 10.1002/ana.410270502 2360787

[B40] Deng X. Berletch J. B. Nguyen D. K. Disteche C. M. (2014). X chromosome regulation: diverse patterns in development, tissues and disease. Nat. Rev. Genet. 15, 367. 10.1038/nrg3687 24733023 PMC4117651

[B41] Diogenes T. C. P. Mourato F. A. de Lima Filho J. L. Mattos S. da S. (2017). Gender differences in the prevalence of congenital heart disease in Down's syndrome: a brief meta-analysis. BMC Med. Genet. 18, 1–5. 10.1186/s12881-017-0475-7 28985718 PMC6389118

[B42] Doran E. Keator D. Head E. Phelan M. J. Kim R. Totoiu M.. (2017). Down syndrome, partial trisomy 21, and absence of Alzheimer's disease: the role of APP. J. Alzheimers. Dis. 56, 459–470. 10.3233/JAD-160836 27983553 PMC5662115

[B43] Dotiwala A. K. McCausland C. Samra N. S. (2021). Anatomy, Head and Neck, Blood Brain Barrier. StatPearls. Available online at: https://www.ncbi.nlm.nih.gov/books/NBK519556/ (accessed February 7, 2022). 30137840

[B44] Downes E. C. Robson J. Grailly E. Abdel-All Z. Xuereb J. Brayne C.. (2008). Loss of synaptophysin and synaptosomal-associated protein 25-kDa (SNAP-25) in elderly Down syndrome individuals. Neuropathol. Appl. Neurobiol. 34, 12–22. 10.1111/j.1365-2990.2007.00899.x 18005332

[B45] Dubal D. B. (2020). Sex difference in Alzheimer's disease: an updated, balanced and emerging perspective on differing vulnerabilities. Handb. Clin. Neurol. 175, 261–273. 10.1016/B978-0-444-64123-6.00018-7 33008530

[B46] Dumanski J. P. Lambert J.-C. Rasi C. Giedraitis V. Davies H. Grenier-Boley B.. (2016). Mosaic loss of chromosome y in blood is associated with Alzheimer disease. Am. J. Hum. Genet. 98, 1208–1219. 10.1016/j.ajhg.2016.05.014 27231129 PMC4908225

[B47] El Hajj N. Dittrich M. Böck J. Kraus T. F. J. Nanda I. Müller T.. (2016). Epigenetic dysregulation in the developing Down syndrome cortex. Epigenetics 11, 563–578. 10.1080/15592294.2016.1192736 27245352 PMC4990229

[B48] Emerson J. F. Kesslak J. P. Chen P. C. Lott I. T. (1995). Magnetic resonance imaging of the aging brain in Down syndrome. Prog. Clin. Biol. Res. 393, 123–138. 8545445

[B49] Engidawork E. Lubec G. (2001). Protein expression in down syndrome brain. Amino Acids 21, 331–361. 10.1007/s007260170001 11858695

[B50] Ferretti M. T. Iulita M. F. Cavedo E. Chiesa P. A. Schumacher Dimech A. Santuccione Chadha A.. (2018). Sex differences in Alzheimer disease—the gateway to precision medicine. Nat. Rev. Neurol. 14, 457–469. 10.1038/s41582-018-0032-9 29985474

[B51] Fiest K. M. Roberts J. I. Maxwell C. J. Hogan D. B. Smith E. E. Frolkis A.. (2016). The Prevalence and incidence of dementia due to Alzheimer's disease: a systematic review and meta-analysis. Can. J. Neurol. Sci. J. Can. des Sci. Neurol. 43, S51–S82. 10.1017/cjn.2016.36 27307128

[B52] Flores-Aguilar L. Iulita M. F. Kovecses O. Torres M. D. Levi S. M. Zhang Y.. (2020). Evolution of neuroinflammation across the lifespan of individuals with Down syndrome. Brain 143, 3653. 10.1093/brain/awaa326 33206953 PMC7805813

[B53] Fortea J. Zaman S. H. Hartley S. Rafii M. S. Head E. Carmona-Iragui M. (2021). Alzheimer's disease associated with Down syndrome: a genetic form of dementia. Lancet. Neurol. 20, 930–942. 10.1016/S1474-4422(21)00245-3 34687637 PMC9387748

[B54] Gaetani L. Blennow K. Calabresi P. Di Filippo M. Parnetti L. Zetterberg H. (2019). Neurofilament light chain as a biomarker in neurological disorders. J. Neurol. Neurosurg. Psychiatry 90, 870 LP−881. 10.1136/jnnp-2018-320106 30967444

[B55] Gaillard R. C. Spinedi E. (1998). Sex- and stress-steroids interactions and the immune system: evidence for a neuroendocrine-immunological sexual dimorphism. Domest. Anim. Endocrinol. 15, 345–352. 10.1016/S0739-7240(98)00028-9 9785038

[B56] Garcia-Segura L. M. Azcoitia I. DonCarlos L. L. (2001). Neuroprotection by estradiol. Prog. Neurobiol. 63, 29–60. 10.1016/S0301-0082(00)00025-3 11040417

[B57] Gilsanz P. Lee C. Corrada M. M. Kawas C. H. Quesenberry C. P. J. Whitmer R. A. (2019). Reproductive period and risk of dementia in a diverse cohort of health care members. Neurology 92, e2005–e2014. 10.1212/WNL.0000000000007326 30923235 PMC6511081

[B58] Goodman Y. Bruce A. J. Cheng B. Mattson M. P. (1996). Estrogens attenuate and corticosterone exacerbates excitotoxicity, oxidative injury, and amyloid beta-peptide toxicity in hippocampal neurons. J. Neurochem. 66, 1836–1844. 10.1046/j.1471-4159.1996.66051836.x 8780008

[B59] Graff-Radford J. Arenaza-Urquijo E. M. Knopman D. S. Schwarz C. G. Brown R. D. Rabinstein A. A.. (2019). White matter hyperintensities: relationship to amyloid and tau burden. Brain 142, 2483–2491. 10.1093/brain/awz162 31199475 PMC6658846

[B60] Griffin W. S. T. Stanley L. C. Ling C. White L. MacLeod V. Perrot L. J.. (1989). Brain interleukin 1 and S-100 immunoreactivity are elevated in Down syndrome and Alzheimer disease. Proc. Natl. Acad. Sci. U. S. A. 86, 7611–7615. 10.1073/pnas.86.19.7611 2529544 PMC298116

[B61] Guaraldi F. Rossetto Giaccherino R. Lanfranco F. Motta G. Gori D. Arvat E.. (2017). Endocrine autoimmunity in Down's syndrome. Front. Horm. Res. 48, 133–146. 10.1159/000452912 28245458

[B62] Hamlett E. D. Goetzl E. J. Ledreux A. Vasilevko V. Boger H. A. LaRosa A.. (2017). Neuronal exosomes reveal Alzheimer's disease biomarkers in Down syndrome. Alzheimers. Dement. 13, 541–549. 10.1016/j.jalz.2016.08.012 27755974 PMC5812672

[B63] Hamlett E. D. Ledreux A. Potter H. Chial H. J. Patterson D. Espinosa J. M.. (2018). Exosomal biomarkers in Down syndrome and Alzheimer's disease. Free Radic. Biol. Med. 114, 110–121. 10.1016/j.freeradbiomed.2017.08.028 28882786 PMC6135098

[B64] Han R. T. Kim R. D. Molofsky A. V. Liddelow S. A. (2021). Astrocyte-immune cell interactions in physiology and pathology. Immunity 54, 211–224. 10.1016/j.immuni.2021.01.013 33567261

[B65] Handen B. L. Cohen A. D. Channamalappa U. Bulova P. Cannon S. A. Cohen W. I.. (2012). Imaging brain amyloid in nondemented young adults with Down syndrome using Pittsburgh compound B. Alzheimers. Dement. 8, 496–501. 10.1016/j.jalz.2011.09.229 23102120 PMC3532743

[B66] Hartley S. L. Handen B. L. Devenny D. Tudorascu D. Piro-Gambetti B. Zammit M. D.. (2020). Cognitive indicators of transition to preclinical and prodromal stages of Alzheimer's disease in Down syndrome. Alzheimer's Dement. 12, e12096–e12096. 10.1002/dad2.12096 PMC7507534 32995465

[B67] Head E. Helman A. M. Powell D. Schmitt F. A. (2018). Down syndrome, beta-amyloid and neuroimaging. Free Radic. Biol. Med. 114, 102–109. 10.1016/j.freeradbiomed.2017.09.013 28935420 PMC5748259

[B68] Head E. Phelan M. J. Doran E. Kim R. C. Poon W. W. Schmitt F. A.. (2017). Cerebrovascular pathology in Down syndrome and Alzheimer disease. Acta Neuropathol. Commun. 5, 93. 10.1186/s40478-017-0499-4 29195510 PMC5709935

[B69] Hebert L. E. Scherr P. A. McCann J. J. Beckett L. A. Evans D. A. (2001). Is the risk of developing Alzheimer's disease greater for women than for men? Am. J. Epidemiol. 153, 132–136. 10.1093/aje/153.2.132 11159157

[B70] Helman A. M. Siever M. McCarty K. L. Lott I. T. Doran E. Abner E. L.. (2019). Microbleeds and cerebral amyloid angiopathy in the brains of people with down syndrome with Alzheimer's disease. J. Alzheimer's Dis. 67, 103–112. 10.3233/JAD-180589 30452414 PMC6424116

[B71] Huang Y. Mahley R. W. (2014). Apolipoprotein E: structure and function in lipid metabolism, neurobiology, and Alzheimer's diseases. Neurobiol. Dis. 72 Pt A, 3–12. 10.1016/j.nbd.2014.08.025 25173806 PMC4253862

[B72] Ishunina T. A. (2021). Alternative splicing in aging and Alzheimer's disease: highlighting the role of tau and estrogen receptor α isoforms in the hypothalamus. Handb. Clin. Neurol. 182, 177–189. 10.1016/B978-0-12-819973-2.00012-5 34266591

[B73] Jaffe A. B. Toran-Allerand C. D. Greengard P. Gandy S. E. (1994). Estrogen regulates metabolism of Alzheimer amyloid beta precursor protein. J. Biol. Chem. 269, 13065–13068. 10.1016/S0021-9258(17)36796-0 8175728

[B74] Kalaria R. N. (1999). The blood-brain barrier and cerebrovascular pathology in Alzheimer's disease. Ann. N. Y. Acad. Sci. 893, 113–125. 10.1111/j.1749-6632.1999.tb07821.x 10672233

[B75] Keator D. B. Phelan M. J. Taylor L. Doran E. Krinsky-McHale S. Price J.. (2020). Down syndrome: distribution of brain amyloid in mild cognitive impairment. Alzheimer's Dement 12, e12013–e12013. 10.1002/dad2.12013 PMC7233421 32435685

[B76] Kimura D. Hampson E. (1994). Cognitive pattern in men and women is influenced by fluctuations in sex hormones. Curr. Dir. Psychol. Sci. 3, 57–61. 10.1111/1467-8721.ep10769964

[B77] Kittler P. Krinsky-McHale S. J. Devenny D. A. (2004). Sex differences in performance over 7 years on the Wechsler Intelligence Scale for Children–Revised among adults with intellectual disability. J. Intellect. Disabil. Res. 48, 114–122. 10.1111/j.1365-2788.2004.00500.x 14723654

[B78] Koedam E. L. G. E. Lauffer V. van der Vlies A. E. van der Flier W. M. Scheltens P. Pijnenburg Y. A. L. (2010). Early-versus late-onset Alzheimer's disease: more than age alone. J. Alzheimer's Dis. 19, 1401–1408. 10.3233/JAD-2010-1337 20061618

[B79] Koran M. E. I. Wagener M. Hohman T. J. Initiative A. N. (2017). Sex differences in the association between AD biomarkers and cognitive decline. Brain Imaging Behav. 11, 205–213. 10.1007/s11682-016-9523-8 26843008 PMC4972701

[B80] Lai F. Kammann E. Rebeck G. W. Anderson A. Chen Y. Nixon R. A. (1999). APOE genotype and gender effects on Alzheimer disease in 100 adults with Down syndrome. Neurology 53, 331. 10.1212/WNL.53.2.331 10430422

[B81] Lai F. Mhatre P. G. Yang Y. Wang M. C. Schupf N. Rosas H. D. (2020). Sex differences in risk of Alzheimer's disease in adults with Down syndrome. Alzheimer's Dement. Diagnosis Assess. Dis. Monit. 12, 1–8. 10.1002/dad2.12084 PMC7507514 32995462

[B82] Landes S. D. Stevens J. D. Turk M. A. (2020). Cause of death in adults with Down syndrome in the United States. Disabil. Health J. 13, 100947. 10.1016/j.dhjo.2020.100947 32680774 PMC7655667

[B83] Landt J. D'Abrera J. C. Holland A. J. Aigbirhio F. I. Fryer T. D. Canales R.. (2011). Using positron emission tomography and carbon 11–labeled pittsburgh compound B to image brain fibrillar β-amyloid in adults with Down syndrome: safety, acceptability, and feasibility. Arch. Neurol. 68, 890–896. 10.1001/archneurol.2011.36 21403005

[B84] Lao P. J. Betthauser T. J. Hillmer A. T. Price J. C. Klunk W. E. Mihaila I.. (2016). The effects of normal aging on amyloid-β deposition in nondemented adults with Down syndrome as imaged by carbon 11-labeled Pittsburgh compound B. Alzheimers. Dement. 12, 380–390. 10.1016/j.jalz.2015.05.013 26079411 PMC4677061

[B85] Lao P. J. Gutierrez J. Keator D. Rizvi B. Banerjee A. Igwe K. C.. (2020). Alzheimer-related cerebrovascular disease in Down syndrome. Ann. Neurol. 88, 1165–1177. 10.1002/ana.25905 32944999 PMC7729262

[B86] Lee N. R. Anand P. Will E. Adeyemi E. I. Clasen L. S. Blumenthal J. D.. (2015). Everyday executive functions in down syndrome from early childhood to young adulthood: evidence for both unique and shared characteristics compared to youth with sex chromosome trisomy (XXX and XXY). Front. Behav. Neurosci. 9, 264. 10.3389/fnbeh.2015.00264 26539087 PMC4611056

[B87] Lenz K. M. McCarthy M. M. (2014). A starring role for microglia in brain sex differences. Neuroscience 21, 306–321. 10.1177/1073858414536468 PMC5742269 24871624

[B88] Li G. Shofer J. B. Petrie E. C. Yu C.-E. Wilkinson C. W. Figlewicz D. P.. (2017). Cerebrospinal fluid biomarkers for Alzheimer's and vascular disease vary by age, gender, and APOE genotype in cognitively normal adults. Alzheimers. Res. Ther. 9, 48. 10.1186/s13195-017-0271-9 28673336 PMC5496132

[B89] Li M. Li Y. Zuo L. Hu W. Jiang T. (2021). Increase of blood-brain barrier leakage is related to cognitive decline in vascular mild cognitive impairment. BMC Neurol. 21, 159. 10.1186/s12883-021-02189-6 33858381 PMC8048027

[B90] Liu C.-C. Kanekiyo T. Xu H. Bu G. (2013). Apolipoprotein E and Alzheimer disease: risk, mechanisms and therapy. Nat. Rev. Neurol. 9, 106–118. 10.1038/nrneurol.2012.263 23296339 PMC3726719

[B91] Lott I. T. Head E. (2019). Dementia in Down syndrome: unique insights for Alzheimer disease research. Nat. Rev. Neurol. 15, 135–147. 10.1038/s41582-018-0132-6 30733618 PMC8061428

[B92] Lv W. Du N. Liu Y. Fan X. Wang Y. Jia X.. (2016). Low testosterone level and risk of Alzheimer's disease in the elderly men: a systematic review and meta-analysis. Mol. Neurobiol. 53, 2679–2684. 10.1007/s12035-015-9315-y 26154489

[B93] Maggioli E. McArthur S. Mauro C. Kieswich J. Kusters D. H. M. Reutelingsperger C. P. M.. (2016). Estrogen protects the blood–brain barrier from inflammation-induced disruption and increased lymphocyte trafficking. Brain. Behav. Immun. 51, 212–222. 10.1016/j.bbi.2015.08.020 26321046

[B94] Marbouti L. Zahmatkesh M. Riahi E. Shafiee Sabet M. (2020). GnRH protective effects against amyloid β-induced cognitive decline: a potential role of the 17β-estradiol. Mol. Cell. Endocrinol. 518, 110985. 10.1016/j.mce.2020.110985 32805333

[B95] Martínez-Cué C. Baamonde C. Lumbreras M. Paz J. Davisson M. T. Schmidt C.. (2002). Differential effects of environmental enrichment on behavior and learning of male and female Ts65Dn mice, a model for Down syndrome. Behav. Brain Res. 134, 185–200. 10.1016/S0166-4328(02)00026-8 12191805

[B96] Martínez-Cué C. Rueda N. García E. Flórez J. (2006). Anxiety and panic responses to a predator in male and female Ts65Dn mice, a model for Down syndrome. Genes. Brain. Behav. 5, 413–422. 10.1111/j.1601-183X.2005.00175.x 16879635

[B97] Matias I. Morgado J. Gomes F. C. A. (2019). Astrocyte heterogeneity: impact to brain aging and disease. Front. Aging Neurosci. 11, 59. 10.3389/fnagi.2019.00059 30941031 PMC6433753

[B98] Matthews F. E. Stephan B. C. M. Robinson L. Jagger C. Barnes L. E. Arthur A.. (2016). A two decade dementia incidence comparison from the Cognitive Function and Ageing Studies I and II. Nat. Commun. 7, 11398. 10.1038/ncomms11398 27092707 PMC4838896

[B99] Mattsson N. Insel P. S. Palmqvist S. Portelius E. Zetterberg H. Weiner M.. (2016). Cerebrospinal fluid tau, neurogranin, and neurofilament light in Alzheimer's disease. EMBO Mol. Med. 8, 1184–1196. 10.15252/emmm.201606540 27534871 PMC5048367

[B100] McCarthy M. Raval A. P. (2020). The peri-menopause in a woman's life: a systemic inflammatory phase that enables later neurodegenerative disease. J. Neuroinflammation 17, 317. 10.1186/s12974-020-01998-9 33097048 PMC7585188

[B101] McEwen B. (2002). Estrogen actions throughout the brain. Recent Prog. Horm. Res. 57, 357–384. 10.1210/rp.57.1.357 12017552

[B102] Mhatre P. G. Lee J. H. Pang D. Zigman W. B. Tycko B. Krinsky-Mchale S. J.. (2021). The association between sex and risk of Alzheimer's disease in adults with Down syndrome. J. Clin. Med. 10, 966. 10.3390/jcm10132966 34279450 PMC8268850

[B103] Mielke M. M. (2020). Consideration of sex differences in the measurement and interpretation of Alzheimer disease-related biofluid-based biomarkers. J. Appl. Lab. Med. 5, 158–169. 10.1373/jalm.2019.030023 31811073 PMC7246149

[B104] Mielke M. M. Syrjanen J. A. Blennow K. Zetterberg H. Skoog I. Vemuri P.. (2019). Comparison of variables associated with cerebrospinal fluid neurofilament, total-tau, and neurogranin. Alzheimers. Dement. 15, 1437–1447. 10.1016/j.jalz.2019.07.009 31668594 PMC6874755

[B105] Mito T. Pereyra P. M. Becker L. E. (1991). Neuropathology in patients with congenital heart disease and Down syndrome. Pediatr. Pathol. 11, 867–877. 10.3109/15513819109065483 1837924

[B106] Montagne A. Nation D. A. Sagare A. P. Barisano G. Sweeney M. D. Chakhoyan A.. (2020). APOE4 leads to blood–brain barrier dysfunction predicting cognitive decline. Nature 581, 71–76. 10.1038/s41586-020-2247-3 32376954 PMC7250000

[B107] Nation D. A. Sweeney M. D. Montagne A. Sagare A. P. D'Orazio L. M. Pachicano M.. (2019). Blood–brain barrier breakdown is an early biomarker of human cognitive dysfunction. Nat. Med. 25, 270–276. 10.1038/s41591-018-0297-y 30643288 PMC6367058

[B108] Neale N. Padilla C. Fonseca L. M. Holland T. Zaman S. (2018). Neuroimaging and other modalities to assess Alzheimer's disease in Down syndrome. NeuroImage. Clin. 17, 263–271. 10.1016/j.nicl.2017.10.022 29159043 PMC5683343

[B109] Nelson P. T. Schmitt F. A. Jicha G. A. Kryscio R. J. Abner E. L. Smith C. D.. (2010). Association between male gender and cortical Lewy body pathology in large autopsy series. J. Neurol. 257, 1875–1881. 10.1007/s00415-010-5630-4 20563821 PMC3040648

[B110] Newcombe E. A. Camats-Perna J. Silva M. L. Valmas N. Huat T. J. Medeiros R. (2018). Inflammation: the link between comorbidities, genetics, and Alzheimer's disease. J. Neuroinflammation 15, 276. 10.1186/s12974-018-1313-3 30249283 PMC6154824

[B111] Oveisgharan S. Arvanitakis Z. Yu L. Farfel J. Schneider J. A. Bennett D. A. (2018). Sex differences in Alzheimer's disease and common neuropathologies of aging. Acta Neuropathol. 136, 887. 10.1007/s00401-018-1920-1 30334074 PMC6279593

[B112] Pentz R. Iulita M. F. Ducatenzeiler A. Videla L. Benejam B. Carmona-Iragui M.. (2021). Nerve growth factor (NGF) pathway biomarkers in Down syndrome prior to and after the onset of clinical Alzheimer's disease: a paired CSF and plasma study. Alzheimers. Dement. 17, 605–617. 10.1002/alz.12229 33226181 PMC8043977

[B113] Petersen M. E. Rafii M. S. Zhang F. Hall J. Julovich D. Ances B. M.. (2021). Plasma total-tau and neurofilament light chain as diagnostic biomarkers of Alzheimer's disease dementia and mild cognitive impairment in adults with down syndrome. J. Alzheimers. Dis. 79, 671–681. 10.3233/JAD-201167 33337378 PMC8273927

[B114] Petersen M. E. Zhang F. Schupf N. Krinsky-McHale S. J. Hall J. Mapstone M.. (2020). Proteomic profiles for Alzheimer's disease and mild cognitive impairment among adults with Down syndrome spanning serum and plasma: an Alzheimer's biomarker consortium-down syndrome (ABC-DS) study. Alzheimer's Dement 12, e12039–e12039. 10.1002/dad2.12039 PMC7327223 32626817

[B115] Podcasy J. L. Epperson C. N. (2016). Considering sex and gender in Alzheimer disease and other dementias. Dialogues Clin. Neurosci. 18, 437–446. 10.31887/DCNS.2016.18.4/cepperson 28179815 PMC5286729

[B116] Popova G. Paterson W. F. Brown A. Donaldson M. D. C. (2008). Hashimoto's thyroiditis in Down's syndrome: clinical presentation and evolution. Horm. Res. 70, 278–284. 10.1159/000157874 18824866

[B117] Prasher V. P. Farrer M. J. Kessling A. M. Fisher E. M. C. West R. J. Barber P. C.. (1998). Molecular mapping of Alzheimer-type dementia in Down's syndrome. Ann. Neurol. 43, 380–383. 10.1002/ana.410430316 9506555

[B118] Rafii M. S. Lukic A. S. Andrews R. D. Brewer J. Rissman R. A. Strother S. C.. (2017). PET imaging of tau pathology and relationship to amyloid, longitudinal mri, and cognitive change in down syndrome: results from the down syndrome biomarker initiative (DSBI). J. Alzheimer's Dis. 60, 439–450. 10.3233/JAD-170390 28946567

[B119] Raghavan R. Khin-Nu C. Brown A. G. Day K. A. Tyrer S. P. Ince P. G.. (1994). Gender differences in the phenotypic expression of Alzheimer's disease in Down's syndrome (trisomy 21). Neuroreport 5, 1393–1396. 10.1097/00001756-199406270-00025 7919207

[B120] Rahman A. Schelbaum E. Hoffman K. Diaz I. Hristov H. Andrews R.. (2020). Sex-driven modifiers of Alzheimer risk: a multimodality brain imaging study. Neurology 95, e166–e178. 10.1212/WNL.0000000000009781 32580974 PMC7455325

[B121] Rajendran L. Paolicelli R. C. (2018). Microglia-mediated synapse loss in Alzheimer's disease. J. Neurosci. 38, 2911–2919. 10.1523/JNEUROSCI.1136-17.2017 29563239 PMC6596066

[B122] Real R. Peter M. Trabalza A. Khan S. Smith M. A. Dopp J.. (2018). *In vivo* modeling of human neuron dynamics and Down syndrome. Science. 362, eaau1810. 10.1126/science.aau1810 PMC6570619 30309905

[B123] Rodrigues R. Debom G. Soares F. Machado C. Pureza J. Peres W.. (2014). Alterations of ectonucleotidases and acetylcholinesterase activities in lymphocytes of Down syndrome subjects: relation with inflammatory parameters. Clin. Chim. Acta. 433, 105–110. 10.1016/j.cca.2014.03.002 24631131

[B124] Safieh M. Korczyn A. D. Michaelson D. M. (2019). ApoE4: an emerging therapeutic target for Alzheimer's disease. BMC Med. 17, 64. 10.1186/s12916-019-1299-4 30890171 PMC6425600

[B125] Santoro M. Coi A. Spadoni I. Bianchi F. Pierini A. (2018). Sex differences for major congenital heart defects in Down Syndrome: a population based study. Eur. J. Med. Genet. 61, 546–550. 10.1016/j.ejmg.2018.05.013 29753092

[B126] Schupf N. Kapell D. Nightingale B. Rodriguez A. Tycko B. Mayeux R. (1998). Earlier onset of Alzheimer's disease in men with Down syndrome. Neurology 50, 991–995. 10.1212/WNL.50.4.991 9566384

[B127] Schupf N. Lee J. H. Pang D. Zigman W. B. Tycko B. Krinsky-McHale S.. (2018). Epidemiology of estrogen and dementia in women with Down syndrome. Free Radic. Biol. Med. 114, 62–68. 10.1016/j.freeradbiomed.2017.08.019 28843780 PMC5748249

[B128] Schupf N. Pang D. Patel B. N. Silverman W. Schubert R. Lai F.. (2003). Onset of dementia is associated with age at menopause in women with Down's syndrome. Ann. Neurol. 54, 433–438. 10.1002/ana.10677 14520653

[B129] Schupf N. Winsten S. Patel B. Pang D. Ferin M. Zigman W. B.. (2006). Bioavailable estradiol and age at onset of Alzheimer's disease in postmenopausal women with Down syndrome. Neurosci. Lett. 406, 298–302. 10.1016/j.neulet.2006.07.062 16926067

[B130] Simpkins J. W. Singh M. Brock C. Etgen A. M. (2012). Neuroprotection and estrogen receptors. Neuroendocrinology 96, 119–130. 10.1159/000338409 22538356 PMC6507404

[B131] Snyder H. M. Asthana S. Bain L. Brinton R. Craft S. Dubal D. B.. (2016). Sex biology contributions to vulnerability to Alzheimer's disease: a think tank convened by the Women's Alzheimer's Research Initiative. Alzheimers. Dement. 12, 1186–1196. 10.1016/j.jalz.2016.08.004 27692800 PMC10341380

[B132] Spampinato S. F. Molinaro G. Merlo S. Iacovelli L. Caraci F. Battaglia G.. (2012). Estrogen receptors and type 1 metabotropic glutamate receptors are interdependent in protecting cortical neurons against β-amyloid toxicity. Mol. Pharmacol. 81, 12–20. 10.1124/mol.111.074021 21984253

[B133] Sperling R. A. Donohue M. C. Raman R. Sun C.-K. Yaari R. Holdridge K.. (2020). Association of factors with elevated amyloid burden in clinically normal older individuals. JAMA Neurol. 77, 735–745. 10.1001/jamaneurol.2020.0387 32250387 PMC7136861

[B134] Stancliffe R. J. Lakin K. C. Larson S. A. Engler J. Taub S. Fortune J.. (2012). Demographic characteristics, health conditions, and residential service use in adults with Down syndrome in 25 U.S. states. Intellect. Dev. Disabil. 50, 92–108. 10.1352/1934-9556-50.2.92 22642964

[B135] Startin C. M. D'Souza H. Ball G. Hamburg S. Hithersay R. Hughes K. M. O.. (2020). Health comorbidities and cognitive abilities across the lifespan in Down syndrome. J. Neurodev. Disord. 12, 4. 10.1186/s11689-019-9306-9 31973697 PMC6979347

[B136] Strydom A. Coppus A. Blesa R. Danek A. Fortea J. Hardy J.. (2018). Alzheimer's disease in Down syndrome: an overlooked population for prevention trials. Alzheimer's Dement. Transl. Res. Clin. Interv. 4, 703–713. 10.1016/j.trci.2018.10.006 PMC6296162 30581976

[B137] Subhadra B. Schaller K. Seeds N. W. (2013). Neuroserpin up-regulation in the Alzheimer's disease brain is associated with elevated thyroid hormone receptor-β1 and HuD expression. Neurochem. Int. 63, 476–481. 10.1016/j.neuint.2013.08.010 24036060 PMC3902180

[B138] Tang M.-X. Jacobs D. Stern Y. Marder K. Schofield P. Gurland B.. (1996). Effect of oestrogen during menopause on risk and age at onset of Alzheimer's disease. Lancet 348, 429–432. 10.1016/S0140-6736(96)03356-9 8709781

[B139] Teipel S. J. Hampel H. (2006). Neuroanatomy of Down syndrome *in vivo*: a model of preclinical Alzheimer's disease. Behav. Genet. 36, 405–415. 10.1007/s10519-006-9047-x 16485178

[B140] Terry R. D. Masliah E. Salmon D. P. Butters N. DeTeresa R. Hill R.. (1991). Physical basis of cognitive alterations in Alzheimer's disease: synapse loss is the major correlate of cognitive impairment. Ann. Neurol. 30, 572–580. 10.1002/ana.410300410 1789684

[B141] Tubi M. A. Feingold F. W. Kothapalli D. Hare E. T. King K. S. Thompson P. M.. (2020). White matter hyperintensities and their relationship to cognition: effects of segmentation algorithm. Neuroimage 206, 116327. 10.1016/j.neuroimage.2019.116327 31682983 PMC6981030

[B142] Tudorascu D. L. Laymon C. M. Zammit M. Minhas D. S. Anderson S. J. Ellison P. A.. (2020). Relationship of amyloid beta and neurofibrillary tau deposition in Neurodegeneration in Aging Down Syndrome (NiAD) study at baseline. Alzheimer's Dement. 6, e12096–e12096. 10.1002/trc2.12096 PMC7602678 33163613

[B143] Weis S. N. Souza J. M. F. Hoppe J. B. Firmino M. Auer M. Ataii N. N.. (2021). In-depth quantitative proteomic characterization of organotypic hippocampal slice culture reveals sex-specific differences in biochemical pathways. Sci. Rep. 11, 2560. 10.1038/s41598-021-82016-7 33510253 PMC7844295

[B144] Whitacre C. C. (2001). Sex differences in autoimmune disease. Nat. Immunol. 2, 777–780. 10.1038/ni0901-777 11526384

[B145] Whitmer R. A. Quesenberry C. P. Zhou J. Yaffe K. (2011). Timing of hormone therapy and dementia: the critical window theory revisited. Ann. Neurol. 69, 163–169. 10.1002/ana.22239 21280086 PMC3058824

[B146] Wilcock D. M. Hurban J. Helman A. M. Sudduth T. L. McCarty K. L. Beckett T. L.. (2015). Down syndrome individuals with Alzheimer's disease have a distinct neuroinflammatory phenotype compared to sporadic Alzheimer's disease. Neurobiol. Aging 36, 2468–2474. 10.1016/j.neurobiolaging.2015.05.016 26103884 PMC4602365

[B147] Wilcock D. M. Schmitt F. A. Head E. (2016). Cerebrovascular contributions to aging and Alzheimer's disease in Down syndrome. Biochim. Biophys. Acta Mol. Basis Dis. 1862, 909–914. 10.1016/j.bbadis.2015.11.007 PMC4821721 26593849

[B148] Xu H. Wang R. Zhang Y.-W. Zhang X. (2006). Estrogen, beta-amyloid metabolism/trafficking, and Alzheimer's disease. Ann. N. Y. Acad. Sci. 1089, 324–342. 10.1196/annals.1386.036 17261779

[B149] Yasin S. R. Tahtamouni L. H. Najeeb N. S. Issa N. M. Al-Mazaydeh Z. A. Alfaouri A. A. (2014). Genomic integrity of the Y chromosome sequence-tagged-sites in infertile and Down syndrome Jordanian males. Andrologia 46, 770–776. 10.1111/and.12147 23957314

[B150] Zammit M. D. Tudorascu D. L. Laymon C. M. Hartley S. L. Ellison P. A. Zaman S. H.. (2021). Neurofibrillary tau depositions emerge with subthreshold cerebral beta-amyloidosis in down syndrome. NeuroImage. Clin. 31, 102740. 10.1016/j.nicl.2021.102740 34182407 PMC8252122

[B151] Zhao L. Woody S. K. Chhibber A. (2015). Estrogen receptor β in Alzheimer's disease: from mechanisms to therapeutics. Ageing Res. Rev. 24, 178–190. 10.1016/j.arr.2015.08.001 26307455 PMC4661108

[B152] Zigman W. B. (2013). Atypical aging in down syndrome. Dev. Disabil. Res. Rev. 18, 51–67. 10.1002/ddrr.1128 23949829

